# Perception of harm is strongly associated with complete ban on in-home cannabis smoking: a cross-sectional study

**DOI:** 10.1186/s12889-024-18072-1

**Published:** 2024-03-02

**Authors:** Osika Tripathi, Humberto Parada, Yuyan Shi, Georg E. Matt, Penelope J. E. Quintana, Sandy Liles, John Bellettiere

**Affiliations:** 1 San Diego State University, School of Public Health, San Diego, California USA; 2 University of California, Herbert Wertheim School of Public Health, San Diego, California USA; 3 San Diego State University, Department of Psychology, San Diego, CA USA

**Keywords:** Cannabis secondhand smoke, Perceived harm, Smoke-free homes

## Abstract

**Background:**

Perception of health risk can influence household rules, but little is known about how the perception of harm from cannabis secondhand smoke (cSHS) is related to having a complete ban on in-home cannabis smoking. We examined this association among a nationally representative sample of United States adults.

**Methods:**

Respondents were 21,381 adults from the cross-sectional Marijuana Use and Environmental Survey recruited from December 2019-February 2020. Perceived harm of cSHS exposure (extremely harmful, somewhat harmful, mostly safe, or totally safe) and complete ban of cannabis smoking anywhere in the home (yes or no) were self-reported. Logistic regression for survey-weighted data estimated covariate-adjusted odds ratios (OR) and 95% confidence intervals (CI) for the association between perceived harm of cSHS and complete ban on in-home cannabis smoking. Stratified subgroup analyses (by cannabis smoking status, cannabis use legalization in state of residence, and children under age 6 living in the home) were conducted to quantify effect measure modification of the association between perception of harm and complete ban.

**Results:**

A complete ban on in-home cannabis smoking was reported by 71.8% of respondents. Eight percent reported cSHS as “totally safe”; 20.5% “mostly safe”; 38.3% “somewhat harmful”; and 33.0% “extremely harmful”. Those who reported cSHS as “extremely harmful” had 6 times the odds of a complete ban on in-home cannabis smoking (OR = 6.0, 95%CI = 4.9–7.2) as those reporting smoking as “totally safe”. The odds of a complete ban were higher among those reporting cSHS as “somewhat harmful” (OR = 2.6, 95%CI = 2.2–3.1) or “mostly safe” (OR = 1.4, 95%CI = 1.2–1.7) vs those reporting cSHS as “totally safe”. In each subgroup of cannabis smoking status, state cannabis use legalization, and children under the age of 6 living in the home, perceived harm was associated with a complete ban on in-home cannabis smoking.

**Conclusions:**

Our study demonstrates perceiving cSHS as harmful is strongly associated with having a complete in-home cannabis smoking ban. With almost a third of US adults perceiving cSHS as at least “mostly safe”, there is strong need to educate the general population about potential risks associated with cSHS exposure to raise awareness and encourage adoption of household rules prohibiting indoor cannabis smoking.

## Background

As of April 2023 in the United States (U.S.), 38 states had legalized medical cannabis use, 23 states had legalized recreational cannabis use [1], and more states are pursuing legalization of cannabis use. Since the mid-2000s, cannabis use has increased among various groups, including parents with children at home, young adults, older adults, and tobacco smokers [2–7]. The most commonly reported method of cannabis use is through combustion [8], which, like tobacco, generates emissions of carcinogens and other toxic substances associated with combustion known to be harmful [9–11]. Similar to tobacco, cannabis secondhand smoke (cSHS) can be inhaled by non-users involuntarily while the smoke persists in the air [12].

Cannabis is frequently allowed to be smoked indoors. In the U.S., 71% of respondents from a sample of Facebook users [13], 59% of cannabis users from a sample of U.S. college students [14], and 76% of Airbnb venues in Colorado [15] all reported allowing cannabis use inside their homes or venues. Fifty-five percent of U.S. cannabis users reported smoking inside their homes in the past 30 days, and 30% reported smoking cannabis daily inside of their homes [16]. This suggests that cSHS exposure to non-smoking residents in the homes of cannabis users may be common.

Research on both short-term and long-term health effects of cSHS is an underdeveloped area of study. While there are major gaps in evidence concerning the long-term consequences of cSHS exposure [17, 18], cannabis smoke has a similar chemical composition to that of tobacco smoke, including many of the same carcinogens and toxic chemicals, such as polycyclic aromatic hydrocarbons, aromatic amines, and ammonia [9–11]. Of available studies, cSHS exposure in children has been positively associated with adverse or problematic physical (respiratory infections and associated emergency care) [19, 20], cognitive (lower scores in verbal and memory domains, attention problems), and behavioral (delinquent behaviors, depressive symptoms) [21–23] health outcomes. Therefore, even non-smokers of cannabis, especially children, may be at risk of cSHS exposure and adverse health problems.

Health-related harm perceptions are fundamental to many health behavior change theories [24]. According to current research, accurate and realistic health risk perceptions are key in motivating behavioral change, and modifying harm perceptions has been shown to effectively alter individual health behaviors [24]. Accurate perception of cSHS exposure as harmful to health could lead to the implementation of rules to ban cannabis smoking inside homes. In tobacco research, perception of tobacco smoke as harmful has been strongly associated with the voluntary adoption of a complete ban on indoor smoking [25]. Setting rules to completely ban in-home cannabis smoking could reduce the amount and frequency of cannabis smoking inside homes, ultimately leading to decreased cSHS exposure and better health outcomes. Households with no ban on indoor tobacco smoking had higher numbers of cigarettes smoked inside of homes compared to households with a complete ban [26], and a complete ban on in-home tobacco smoking was associated with lower urinary cotinine levels among children [27]. While few data are available on the relationship between the perception of harm of cSHS exposure and health behavior change, one study among young adults reported that perception of harm from cannabis smoke or vapor byproducts was inversely associated with allowing cannabis use inside of homes [28]. With only half of U.S. adults perceiving cSHS as harmful [29], it is important to address the gaps in understanding how the perception of harm and other covariates affect household rules on in-home cannabis smoking.

In this study, we quantified the association between perceived harm of being exposed to cSHS and household rules on in-home cannabis smoking. We also evaluated effect measure modification by cannabis smoking status, tobacco smoking status, state cannabis legalization, and children (0–5, 6–12, 13–17 years old) residing in the home. We considered these variables as effect measure modifiers as studies have shown that recent cannabis and tobacco use [30] and permissive cannabis laws [31] are associated with the perception of cSHS exposure as harmful. Also, cannabis use, tobacco use, and perceived social acceptability of cannabis usage are negatively related to having household rules on in-home cannabis smoking [28]. We hypothesized that a greater perception of harm from cSHS exposure would be associated with having household rules to ban in-home cannabis smoking.

## Methods

### Sample selection

This cross-sectional study used data from the U.S. Marijuana Use and Environmental Survey (MUES) 2020 collected between December 2019 and February 2020 [8]. MUES 2020 recruited 21,903 adult (18 years or older) respondents from the address-based and probability-based online panel, KnowledgePanel™. This panel has been used to provide representative statistics on drug use for 97% of adults in the U.S. general population in all 50 states and Washington, DC. Survey weights are provided to ensure that the results are representative of the U.S. general population. Patterns in missing data were assessed, but as missingness was low across variables (≤ 1% missing), we conducted a complete case analysis (*n* = 21,381), excluding 522 respondents with missing data.

MUES data were de-identified, and this study was a secondary analysis of the data. Thus, this study does not constitute human subjects research and was therefore not subject to IRB review.

### Measures

#### Perceived harm

Perceived harm of cSHS was measured with the following question: “How harmful do you think it is to be exposed to *secondhand* smoke at least *3 times per* week from the following substance: Marijuana smoke?” The four response options were: “Extremely harmful”, “Somewhat harmful”, “Mostly safe”, and “Totally safe”. A similar measure has been used by the National Adult Tobacco Survey [32].

#### Household rules

Household rules on in-home cannabis smoking were measured with the following question: “Which statement best describes the rules of *smoking* marijuana *inside your home?”* A binary variable was created from four response options: complete ban (“No one is allowed anywhere”) or no complete ban (“Allowed in some places”, “Allowed everywhere”, or “Did not make rules”). A similar measure has been used by the Global Adult Tobacco Survey, and just as in that study, we did not provide definitions for each response option and instead let survey respondents select the one that best matched their household rules [33].

#### Covariates

Demographic variables were self-reported including: sex (male, female); age (continuous in years); race/ethnicity (non-Hispanic White, non-Hispanic Black, Hispanic, non-Hispanic other race, non-Hispanic multiple races); marital status (married, widowed, divorced/separated, never married, living with a partner); education (less than high school, high school, some college, bachelor’s degree or higher); and household income [high (> $99,999), medium ($40,000—$99,999), low (< $40,000)]. Other covariates included: One or more residents of the participant’s home was a child under the age of 6 (yes, no); 6–12 years old (yes, no); 13–17 years old (yes, no); state legalization of cannabis use at time of questionnaire (recreational and medical legalization, only medical, no cannabis legalization); frequency of cannabis smoking in the past 12 months [never smoked cannabis, did not smoke cannabis in the past 12 months (former cannabis smoker), smoked cannabis in the past 12 months (past-year cannabis smoker)]; frequency of cigarette smoking in past 12 months [never smoked cigarettes, did not smoke cigarettes in the past 12 months (former cigarette smoker), smoked cigarettes in the past 12 months (past-year cigarette smoker)]; use of any of the following drugs: opioids, amphetamines, 3,4-methylenedioxy-methamphetamine (MDMA), hallucinogens, heroin, or cocaine (never used any of these drugs; did not use any of these drugs in past 12 months; did not use any of these drugs in past 30 days; used one or more of these drugs in past 30 days).

### Statistical analysis

Descriptive statistics for the study sample with survey weights applied (target population) were stratified by level of perceived harm of cSHS exposure. We used multivariable logistic regression for survey-weighted data to estimate odds ratios (ORs) and corresponding 95% confidence intervals (CIs) of a complete in-home cannabis smoking ban (vs no complete ban) in association with the perceived harm of cSHS exposure. We conducted sequential modeling to examine the extent of confounding by various groups of covariates: Model 1 was an unadjusted model; the only independent variable was perceived harm. Model 2 included Model 1 and demographic variables (age, sex, race/ethnicity, marital status, highest level of education, and household income). Model 3 included Model 2 and cannabis smoking status. Model 4 included Model 3 and tobacco smoking status and use of other drugs. Model 5 included Model 4 and state cannabis legalization. Model 6 included Model 5 and children living in the home. We examined effect measure modification of the relationship between perception of harm and complete ban by cannabis smoking status (never, former, or past-year cannabis smoker), cigarette use (never, former, or past-year cigarette smoker), state cannabis legalization (recreational and medical, only medical, or no cannabis legalization), living with a child under the age of 6 (yes, no), 6–12 years old (yes, no), or 13–17 years old (yes, no) by adding statistical interaction terms in fully-adjusted logistic regression models (i.e., Model 6). Logistic regression models stratified by each statistically significant (*p* < 0.05) effect modifier variable were conducted, and results for each stratum are presented. Before testing all interactions, sample sizes in all strata were confirmed to be sufficiently large (> 10) to provide meaningful analysis. After weighting, all estimates were representative of the U.S. general population.

We conducted additional analyses with the original household rules variable, which had four categories as the outcome variable (“No one is allowed anywhere”, “Allowed in some places”, “Allowed everywhere”, “Did not make rules”). The proportional odds assumption for ordinal regression was not met for any model. Therefore, a multinomial regression was run for the fully-adjusted model, with a complete ban (“No one is allowed anywhere”) as the reference.

 All data management was conducted in R (version 4.0.0; R Foundation for Statistical Computing, Vienna, Austria), and statistical analyses were conducted in SAS Studio (SAS Institute Inc., Cary, NC, U.S.).

## Results

About half of the target population were female (51.8%), and most were non-Hispanic White (64.0%), married (56.9%), had some college education or higher (62.0%), and had middle or high income (73.7%). The average age was 48.2 (standard error = 0.1 years). Less than 15% had children 0–5 years old (11.9%), 6–12 years old (14.2%), or 13–17 years old (12.7%) as household members. Approximately half had never smoked marijuana (49.9%), 33.8% were former cannabis smokers, and 16.3% were past-year cannabis smokers (Table 1).

**Table 1:** Descriptive statistics of the weighted study population (target population) stratified by perceived harm of cannabis secondhand smoke exposure; MUES 2020

Characteristics	Total	Totally safe	Mostly safe	Somewhat harmful	Extremely harmful
	21,318	1,741	4,364	8,184	7,029
	n (%)	n (%)	n (%)	n (%)	n (%)
Complete ban on in-home cannabis smoking					
Yes	15298 (71.8)	640 (36.8)	2354 (53.9)	6056 (74.0)	6248 (88.9)
No	6020 (28.2)	1101 (63.2)	2010 (46.1)	2128 (26.0)	7819 (11.1)
Age					
Mean (Standard error)	48.2 (0.1)	41.2 (0.5)	45.9 (0.3)	48.9 (0.2)	50.5 (0.3)
Sex					
Female	11041 (51.8)	812 (46.6)	2027 (46.4)	4052 (49.5)	4150 (59.0)
Male	10277 (48.2)	929 (53.4)	2337 (53.6)	4132 (50.5)	2879 (41.0)
Race/Ethnicity					
non-Hispanic White	13649 (64.0)	1040 (59.8)	3047 (69.8)	5579 (68.2)	3983 (56.7)
non-Hispanic Black	2461 (11.5)	330 (18.9)	493 (11.3)	844 (10.3)	795 (11.3)
non-Hispanic other race	1400 (6.6)	63 (3.6)	206 (4.7)	566 (6.9)	564 (8.0)
Hispanic	3418 (16.0)	255 (14.6)	526 (12.1)	1056 (12.9)	1582 (22.5)
non-Hispanic multiple race	389 (1.8)	53 (3.1)	92 (2.1)	139 (1.7)	105 (1.5)
Marital status					
Married	12130 (56.9)	677 (38.9)	2211 (50.7)	4809 (58.8)	4433 (63.1)
Widowed	940 (4.4)	40 (2.3)	158 (3.6)	365 (4.5)	377 (5.3)
Divorced/Separated	2424 (11.4)	246 (14.1)	552 (12.6)	870 (10.6)	4433 (63.1)
Never married	4341 (20.4)	469 (27.0)	1012 (23.2)	1642 (20.0)	1218 (17.3)
Living with partner	1483 (6.9)	309 (17.7)	431 (9.9)	498 (6.1)	246 (3.5)
Highest level of education					
Less than high school	2281 (10.7)	278 (16.0)	438 (10.0)	602 (7.4)	963 (13.7)
High school	5816 (27.3)	588 (33.8)	1142 (26.2)	2058 (25.1)	2029 (28.9)
Some college	6509 (30.5)	552 (31.7)	1432 (32.8)	2539 (31.0)	1986 (28.2)
Bachelors or higher	6712 (31.5)	323 (18.5)	1352 (31.0)	2985 (36.5)	2052 (29.2)
Household income					
Low	5605 (26.3)	738 (42.4)	1095 (25.1)	1727 (21.1)	2045 (29.1)
Middle	10678 (50.1)	784 (45.0)	2281 (52.3)	4194 (51.2)	3419 (48.6)
High	5035 (23.6)	218.2 (12.5)	988 (22.6)	2263 (27.7)	1565 (22.2)
Household members aged 0 -5 years old					
Yes	2546 (11.9)	263 (15.1)	541 (12.4)	873 (10.7)	869 (12.4)
No	18772 (88.1)	1478 (84.9)	3823 (87.6)	7311 (89.3)	6160 (87.6)
Household members aged 6–12 years old					
Yes	3037 (14.2)	264 (15.2)	570 (13.1)	1070 (13.1)	1134 (16.1)
No	18281 (85.8)	1477 (84.8)	3794 (86.9)	7714 (86.9)	5896 (83.9)
Household members aged 13–17 years old					
Yes	2710 (12.7)	177 (10.2)	494 (11.3)	961 (11.7)	1079 (15.3)
No	18608 (87.3)	1564 (89.8)	3870 (88.7)	7223 (88.3)	5951 (84.6)
Cannabis legalization in state of residence					
Only medical	8419 (39.5)	656 (37.6)	1823 (41.8)	3227 (39.4)	2713 (38.6)
Medical and recreational	5988 (28.1)	450 (25.9)	1156 (26.5)	2340 (28.6)	2042 (29.0)
Not legal	6911 (32.4)	635 (36.5)	1385 (31.7)	2617 (32.0)	2274 (32.4)
Cannabis smoking status					
Never smoked cannabis	10633 (49.9)	244 (14.0)	1158 (26.6)	4050 (49.5)	5180 (73.7)
Former cannabis smoker	7204 (33.8)	598 (34.3)	1865 (42.7)	3112 (38.0)	1630 (23.2)
Past-year cannabis smoker	3481 (16.3)	899 (51.7)	1341 (30.7)	1022 (12.5)	219 (3.1)
Cigarette smoking status					
Never smoked cigarettes	10193 (47.8)	543 (31.2)	1528 (35.0)	3782 (46.2)	4341 (61.7)
Former cigarette smoker	8177 (38.4)	598 (34.4)	1908 (43.7)	3441 (42.0)	2230 (31.7)
Past-year cigarette smoker	2948 (38.3)	560 (34.4)	928 (21.3)	962 (11.8)	458 (6.5)
Drug use status					
Never used drugs	12128 (56.9)	830 (47.7)	2023 (46.4)	4495 (54.9)	4779 (68.0)
No drug use in 12 months	6498 (30.5)	565 (32.5)	1611 (36.9)	2705 (33.1)	1617 (23.0)
No drug use in past 30 days	1285 (6.0)	133 (7.6)	323 (7.4)	502 (6.1)	328 (4.7)
Used drugs in past 30 days	1407 (6.6)	213 (12.2)	407 (9.3)	482 (5.9)	305 (4.3)

Most (71.4%) of the target population perceived cSHS exposure as harmful (33.0% extremely harmful and 38.4% somewhat harmful), with 28.6% reporting cSHS exposure as safe (20.5% as mostly safe; and 8.1% as totally safe) (Table 1).

Among the target population, 71.8% reported a complete ban on in-home cannabis smoking. The remainder (28.2%) did not have a complete ban, with 9.0% reporting allowing cannabis smoking in some places inside the home, 3.7% allowing it everywhere, and 15.6% not having any rules.

### Sequential modeling results

The odds of a complete ban among those who reported cSHS as “extremely harmful” decreased from 12.1 (95% CI = 10.2–14.4) to 5.0 (95% CI = 5.0–7.2) after adjusting for cannabis smoking status (Table 2; Model 2 vs. Model 3). In the final model, after adjusting for all confounders (Model 6), respondents who reported cSHS as “extremely harmful” had statistically significantly higher odds (OR = 6.0, 95%CI = 4.9–7.2) of having a complete in-home cannabis smoking ban compared to those who reported it as “totally safe” (Table 2). There was a dose–response relationship between level of perceived harm and having a complete ban on in-home cannabis smoking: “extremely harmful” (OR = 6.0, 95%CI = 4.9–7.2), “somewhat harmful” (OR = 2.6, 95%CI = 2.2–3.1), and “mostly safe” (OR = 1.4, 95%CI = 1.2–1.7); compared to those who reported cSHS exposure as “totally safe” (Table 2).

**Table 2:** Sequential modeling of the association between perceived harm of cannabis secondhand smoke exposure and complete ban on in-home cannabis smoking with “no complete ban” as reference

Perception of Harm	OR (95% CI)
Model 1:	
Totally safe	1.0 (ref)
Mostly safe	2.0 (1.7, 2.3)
Somewhat harmful	4.9 (4.2, 5.7)
Extremely harmful	13.8 (11.7, 16.2)
Model 2:	
Totally safe	1.0 (ref)
Mostly safe	1.8 (1.5, 2.1)
Somewhat harmful	4.2 (3.6, 4.9)
Extremely harmful	12.1 (10.2, 14.4)
Model 3:	
Totally safe	1.0 (ref)
Mostly safe	1.4 (1.2, 1.7)
Somewhat harmful	2.6 (2.2, 3.1)
Extremely harmful	6.0 (5.0, 7.2)
Model 4:	
Totally safe	1.0 (ref)
Mostly safe	1.4 (1.2, 1.7)
Somewhat harmful	2.6 (2.2, 3.1)
Extremely harmful	5.9 (4.9, 7.1)
Model 5:	
Totally safe	1.0 (ref)
Mostly safe	1.5 (1.2, 1.7)
Somewhat harmful	2.6 (2.2, 3.1)
Extremely harmful	6.1 (5.0, 7.3)
Model 6:	
Totally safe	1.0 (ref)
Mostly safe	1.4 (1.2, 1.7)
Somewhat harmful	2.6 (2.2, 3.1)
Extremely harmful	6.0 (5.0, 7.2)

Model 1: Unadjusted

Model 2: Adjusted for demographic variables (age, sex, race/ethnicity, marital status, highest level of education, and household income)

Model 3: Adjusted for Model 2 variables + cannabis smoking status

Model 4: Adjusted for Model 3 + cigarette smoking status and drug use status

Model 5: Adjusted for Model 4 + cannabis legalization

Model 6: Adjusted for Model 5 + household members aged 0–5 years, 6–12 years, and 13–17 years

### Effect modification results

**Fig. 1:**
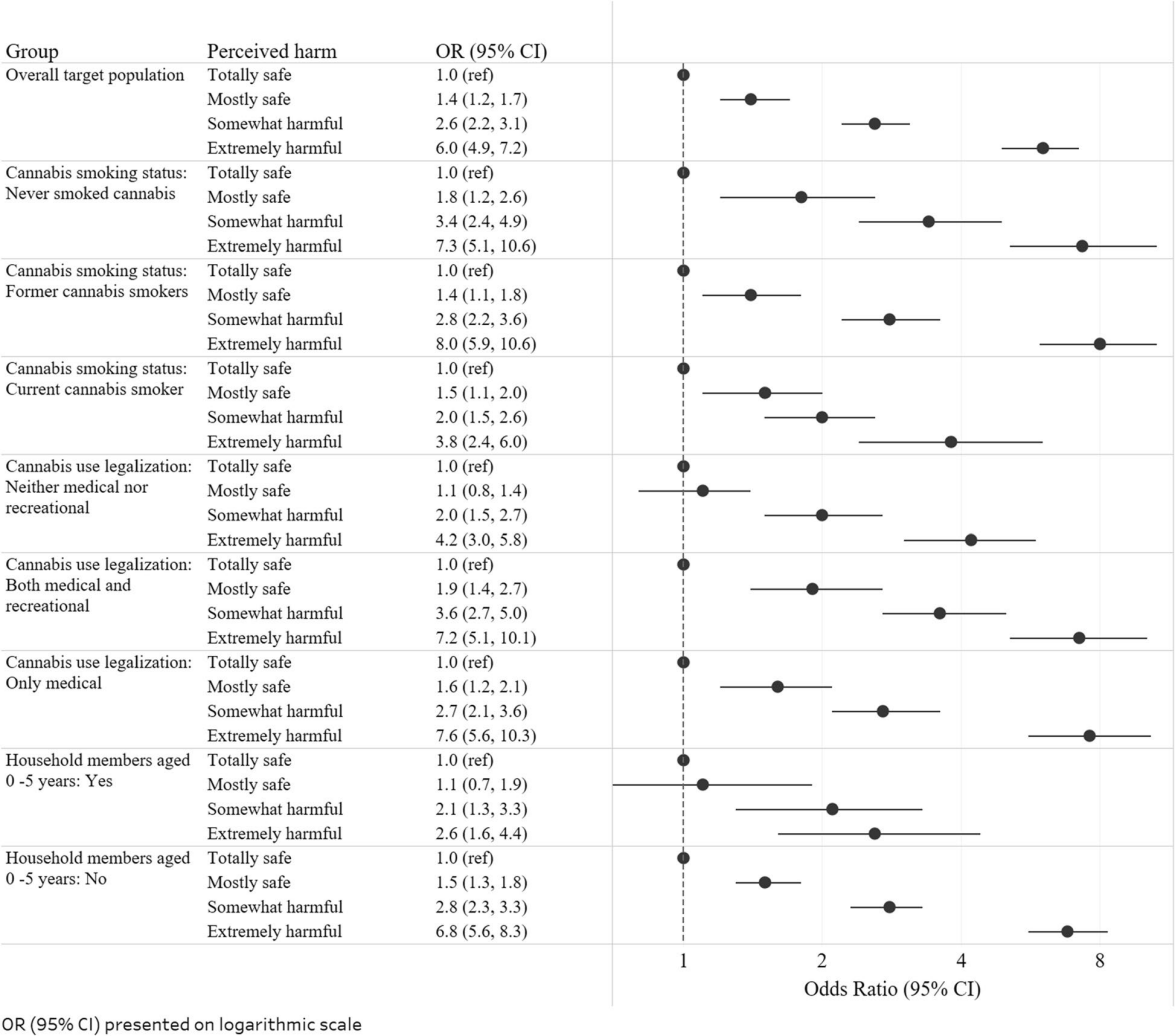
Association between perceived harm of cannabis secondhand smoke exposure and complete ban on in-home cannabis smoking: overall and stratified by effect measure modifiers

Comparing “extremely harmful” to “totally safe” responses, statistically significant interactions (*p* < 0.05) were observed between harm perception and 1) each level of respondents’ cannabis smoking status, 2) each category of state cannabis legalization, and 3) presence vs. absence of children 0–5 years old living in the respondents’ home—demonstrating that the strength of the perceived harm and home smoking ban relationship differed for each level of these effect measure modifiers (Fig. 1). In every sub-group by cannabis smoking status, respondents who reported cSHS as “extremely harmful” had higher odds of having a complete ban on in-home cannabis smoking compared to those who reported it as “totally safe”. Among those who never smoked cannabis and among former cannabis smokers, the odds of having a complete ban on in-home cannabis smoking (vs. no complete ban) were higher than among past-year cannabis smokers (p-interaction = 0.002): among never cannabis smokers (OR = 7.3, 95%CI = 5.1–10.6); among former cannabis smokers (OR = 8.0, 95%CI = 6.0–10.6); and among past-year cannabis smokers (OR = 3.8, 95%CI = 2.4–6.0). In each sub-group of cannabis smoking status, there was a dose–response relationship between levels of perceived harm and having a complete ban on in-home cannabis smoking.

The same pattern was observed for states’ cannabis legalization status (p-interaction = 0.022). The odds (95%CI) of having a complete ban among respondents reporting cSHS as “extremely harmful” vs. those reporting it as “totally safe” were: 7.6 (5.6–10.3) for those living in a state with only medical cannabis use legalized; 7.2 (5.1–10.1) for those in states with both medical and recreational cannabis use legalized; and 4.2 (3.0–5.8) for those in states with neither medical nor recreational cannabis use legalized (Fig. 1). Both among those who reported having household members 0–5 years old and among those who did not, respondents who reported cSHS as “extremely harmful” had statistically significant higher odds of having a complete ban on in-home cannabis smoking compared to those who reported it as “totally safe” (Fig. 1). However, among those who did not have household members 0–5 years old, the odds (OR = 6.8, 95%CI = 5.6–8.3) were significantly higher (p-interaction = 0.002) than among those who did have children 0–5 living in their home (OR = 2.6, 95%CI = 1.6–4.4). In both sub-groups, there was a strong dose–response relationship between levels of perceived harm and having a complete ban on in-home cannabis smoking.

### Multinomial analysis results

After adjusting for all covariates, reporting any level of perceived harm increased the odds of having any level of in-home cannabis smoking rules (allowed everywhere, allowed in some places, no rules) relative to a complete ban (Table 3).

**Table 3:** Multinomial logistic regression of the association between perceived harm of cannabis secondhand smoke exposure and household rules on in-home cannabis smoking with complete ban as reference

	allowed some places vs. complete ban	Did not make rules* vs. complete ban	allowed everywhere vs. complete ban	
	OR (95% CI)	OR (95% CI)	OR (95% CI)	*p*-value
**Overall Final Model**				
* Perceived harm*				< 0.001
Totally safe	1.0 (ref)	1.0 (ref)	1.0 (ref)	
Mostly safe	0.9 (0.7, 1.1)	0.7 (0.6, 0.9)	0.5 (0.4, 0.7)	
Somewhat harmful	0.5 (0.4, 0.7)	0.4 (0.3, 0.5)	0.2 (0.2, 0.3)	
Extremely harmful	0.2 (0.2, 0.3)	0.2 (0.1, 0.2)	0.1 (0.1, 0.2)	

## Discussion

We demonstrate that in the U.S. adult population, the perception of cSHS harm is a key factor related to having a complete in-home cannabis smoking ban. This was true for the whole target population and, with only two exceptions, in every subgroup examined (see Fig. 1). In nearly all analyses, the perception of harm of cSHS exposure at any level more than totally safe was associated with higher odds of having a complete ban on in-home cannabis smoking than of having no complete ban. One subgroup result of note is the three-fold higher odds of a complete ban on in-home cannabis smoking among past-year cannabis smokers who reported cSHS exposure as extremely harmful vs. totally safe. It is evident that even in households with past-year cannabis smokers, perception of harm is highly positively associated with the presence of a complete ban.

There are very few studies, to our knowledge, that have examined the relationship between the perception of harm of cSHS exposure and a complete ban on in-home cannabis smoking. In one study among young adults (ages 18–34) in the U.S., recruited from social media websites, the likelihood of allowing cannabis use in the home was inversely but not significantly associated with the perception of harm from byproducts of cannabis smoke or vapor (beta = -0.03 (95% CI: -0.07, 0.00) [28]. Similar to the estimated 28% in our study who reported cSHS exposure as totally or mostly safe, a 2018 study focused on the perception of cSHS exposure with respondents from KnowledgePanel® reported an estimated 32% of U.S. adults perceived exposure to cSHS as not at all or a little harmful [29], with younger age, recent cannabis use, recent tobacco use, cannabis, and tobacco co-use, and non-White race/ethnicity being positively related to increased likelihood of perceiving cSHS exposure as not harmful [29]. In studies examining household rules, cannabis smoking was more often allowed inside the homes of young adults, cannabis smokers, tobacco smokers, and cannabis and tobacco co-users than inside the homes of non-smokers [13, 14]. Additionally, in homes of young adults, peer use and perceived social acceptability of using cannabis were positively correlated with allowing cannabis use inside homes [28].

In our study, even among past-year cannabis smokers, who are the most likely to put non-smoker residents of their homes at risk of cSHS exposure, the association between perceiving cSHS as harmful and the presence of a complete ban versus not having a complete ban was strong. Past-year cannabis smokers who perceived cSHS as harmful had a three-fold increase in the odds of having a complete ban, compared to a seven-fold increase in the odds among never-cannabis smokers and an eight-fold increase in the odds among former cannabis smokers. The differences in the relationship may be due to skewed perceptions that past-year cannabis smokers have about harms related to cannabis smoke; cannabis smokers perceive cannabis as less addictive and a “healthier” alternative to smoking tobacco [34]. The perception of cSHS as not harmful has been associated with recent cannabis use [29] as well as regular cannabis use [30]. Additionally, past-year cannabis smokers are unlikely to restrict cannabis smoking inside their own homes, as cannabis users have a high (50%) prevalence of in-home cannabis smoking [16]. Educating past-year cannabis smokers on the harms and risks of cannabis smoke could substantially reduce cSHS exposure, as 40% of those without a ban on in-home cannabis smoking were past-year cannabis smokers.

Perception of harm of cSHS exposure was strongly related to a complete ban on in-home cannabis smoking among respondents with and without children under 5 years living in the home. But unexpectedly, among these respondents, for those with children under 5 there was a weaker association than among those without children under 5 (ORs: 2.6 vs. 6.8). These differences may be due to other drivers of the behavior of parents or those with children living at home. Tobacco SHS studies show that while knowledge of risk and perception of harms are important factors, social norms (communities where the high value placed on social relationships makes changing a guest’s behavior difficult and where smoking functions as a positive shared activity), gender imbalances (women’s lack of agency in affecting rules and others’ behavior), and structural factors (living in someone else’s home, such as parent’s house, so cannot establish household rules) are also barriers to smoke-free homes [35]. Understanding other important drivers is important for cannabis smoke-free home efforts, as decreasing or eliminating cSHS exposure of children could greatly impact children’s health outcomes [19, 21–23].

Lastly, among respondents living in states where cannabis use was not legalized, the odds of having a complete ban among those reporting cSHS as “extremely harmful” (ref: “totally safe”) was lower than among respondents living in states where medical or recreational cannabis use was legalized (ORs: 4.2 vs. 7.6 vs. 7.2). While all three associations are strong and statistically significant, the differential odds by cannabis legalization status may be due to various reasons not measured in our study such as ideological and cultural differences, access to educational resources about cannabis, and differing magnitudes of social desirability bias pertaining to answering questions about cannabis smoking perceptions or behaviors. To our knowledge, there are no studies on reasons cannabis legalization status may affect the relationship between perception of harm and complete ban of in-home cannabis smoking. Future studies are needed, to replicate the results by cannabis use legalization. Replication of our results and the causality of the relationship would indicate that in states where medical or recreational cannabis is legalized, campaigns that aim to shift perception of harm of cSHS will have a strong impact on residents to make changes such as instilling household rules on in-home cannabis smoking. Future studies advancing our understanding of how cannabis legalization status might influence public health campaigns to eliminate in-home cannabis smoking are also needed.

Our study had several noteworthy strengths. The large representative sample and low levels of missing data strengthen the external validity of our findings and allow us to examine effect measure modifications by important covariates. The low level of missingness in our data indicates that selection bias was unlikely in our study, conserving the internal validity of our study findings. Our question from MUES on perceived harm of cSHS exposure asks about the degree of harm from exposure to cannabis smoke at least three times per week. There is no standard measurement of the health risk of cSHS, but surveys such as the National Survey on Drug Use and Health use “once or twice a week” to measure the perceived risk from *smoking* cannabis [36]. The frequency of “at least three times per week” used in MUES captures more frequent exposure to cSHS, which may be a cause of greater concern for the general population as compared to occasional cSHS exposure (e.g., once a week or once a month).

Limitations of our study included the cross-sectional study design, which provides no information concerning the temporal order of the associated variables, precluding inferences about causality. However, while it is plausible that the implementation of in-home rules for cannabis smoking could cause someone to perceive cSHS exposure as harmful, this seems unlikely. While our study leads us to recommend promoting the adoption of household rules by residents to reduce indoor cannabis smoke, our study is not able to distinguish if the household rules were imposed by the residents of the home or if they were imposed by owners of a rental home or by policies enacted by state or local agencies. This inability to distinguish the source of rules warrants additional investigation in subsequent studies as it may lead to overestimation of the relationship between perceived harm and household rules. The self-reported nature of our data poses another limitation: participants may under-report sensitive information, such as cannabis use, rules related to cannabis use, or use of other (particularly illegal) drugs. Additionally, household rules on in-home cannabis smoking may mediate the relationship between the perception of harm of cSHS exposure and in-home cannabis smoking behavior. Despite limitations, our study’s focused, detailed look at that relationship provides a solid base for future research exploring its mediation by in-home cannabis smoking behavior.

## Conclusions

Perceived harm from cSHS exposure was strongly associated with a complete ban on in-home cannabis smoking in this nationally representative study of U.S. adults. The odds of having a complete ban on in-home cannabis smoking increased with the perception of harm from cSHS as more harmful, even among past-year cannabis smokers. Thus, promoting a more widespread understanding that cSHS is harmful may facilitate implementation of smoke-free home policies [37]. Creating additional strategies to eliminate indoor cannabis smoking, such as identifying alternative locations outside the home for smoking, should also be explored. The most impact may be achieved through multilevel efforts: changing individual knowledge and increasing peer and community pressure and norms [38].

## Data Availability

Data for this study are available for research upon reasonable request to Dr. Yuyan Shi (yus001@ucsd.edu).

## Abbreviations

CI: Confidence interval
cSHS: Cannabis secondhand smoke
OR: Odds Ratio
U.S.: United States of America
